# Long-term outcomes of modular metal prosthesis replacement in patients with irreparable radial head fractures

**DOI:** 10.1186/s13018-018-0844-8

**Published:** 2018-06-01

**Authors:** Alvin Chao-Yu Chen, Ying-Chao Chou, Chun-Jui Weng, Chun-Ying Cheng

**Affiliations:** Bone and Joint Research Center, Department of Orthopaedic Surgery, Chang Gung Memorial Hospital–Linkou and Chang Gung University College of Medicine, 5th, Fu-Shin Street, Kweishan District, Taoyuan, 333 Taiwan, Republic of China

**Keywords:** Radial head fracture, Radial head replacement, Prosthesis, Radiolucency

## Abstract

**Background:**

The purposes of this study were to investigate the long-term outcomes of radial head replacement and to analyze the relationship between functional outcomes and periprosthetic radiolucency.

**Methods:**

We retrospectively reviewed 32 patients who underwent unilateral radial head replacement between 2004 and 2011. Data on patient characteristics including age, gender, injury complexity, associated trauma, injury chronicity, and number of surgeries were collected and analyzed. Of these patients, 14 had terrible triad injury, 14 valgus-type injuries, 3 Monteggia fracture, and 1 concomitant distal humerus fracture. Clinical survey was performed at 7 to 15 years after replacement surgery. The Mayo Elbow Performance Score (MEPS) and shortened Disabilities of the Arm, Shoulder, and Hand (quickDASH) score were used for functional evaluation. Residual elbow or forearm pain was evaluated using visual analog scale (VAS). Radiographs were reviewed by orthopedic and radiologic specialists, and periprosthetic radiolucency was measured based on the diameter of radial head prosthesis.

**Results:**

The 32 patients returned for follow-up at an average of 8.94 years. None underwent prosthesis revision or removal. MEPS averaged 83.4; good or excellent results were achieved in 26 patients. QuickDASH scores averaged 11.7. Significantly better MEPS (*p* = 0.023) and quickDASH scores (*p* = 0.026) were noted when replacement surgery served as the primary surgery instead of late salvage. VAS scores averaged 1.25, with residual pain noted in 24 elbows (75%). Periprosthetic radiolucency was noted in 21 patients (66%) with a mean thickness of 3.53 mm. The difference in functional outcomes was not significant between patients with and without radiolucency, with *p* values of 0.127 for MEPS and 0.135 for quickDASH scores. Spearman correlation analysis showed low correlation between the measured width of radiolucency and VAS scores (*r* = 0.143).

**Conclusion:**

Sustained, encouraging clinical outcomes were reported in the present study. Although periprosthetic radiolucency did not correlate with functional or pain scores, surgical optimization and meticulous survey were warranted.

## Background

As up to 60% of the force transmits across the radiocapitellar joint [1], the radial head plays an important role in elbow mechanics [2] and serves as a secondary constraint to valgus axial strain [3]. Radial head fractures constitute 33–75% of all elbow fractures [4]. Increasing fracture complexity according to the Mason classification is often associated with complex lesions that also affect the ligamentous structures or coronoid process [5–7]. The critical role played by the radial head in the overall stability of the elbow and high incidence of associated injuries has motivated many orthopedic surgeons to preserve the radial head during fracture treatment [8]. In dealing with comminuted Mason type III radial head fractures that are considered irreparable, radial head replacement (RHR) offers better results than excision alone [9] by restoring elbow kinematics and stability similar to those of the native elbow [10].

With the introduction of radial head prostheses in 1941 [11], various designs were available with respect to material, fixation technique, modularity, and polarity. Among those designs, modular monopolar prostheses with loose-fit implantation became commonly adopted since 2005 [12]. Satisfactory clinical outcomes have been reported in our previous study [13] and recent publications [14–16]. Given the general popularity and favorable outcomes in those studies, long-term data based on both clinical and radiographic survey are yet to be established. The purpose of our study was to report long-term results with RHR and to analyze the effect of radiolucency on clinical outcomes through a quantitative measurement.

## Methods

### Patient data

**Fig. 1:**
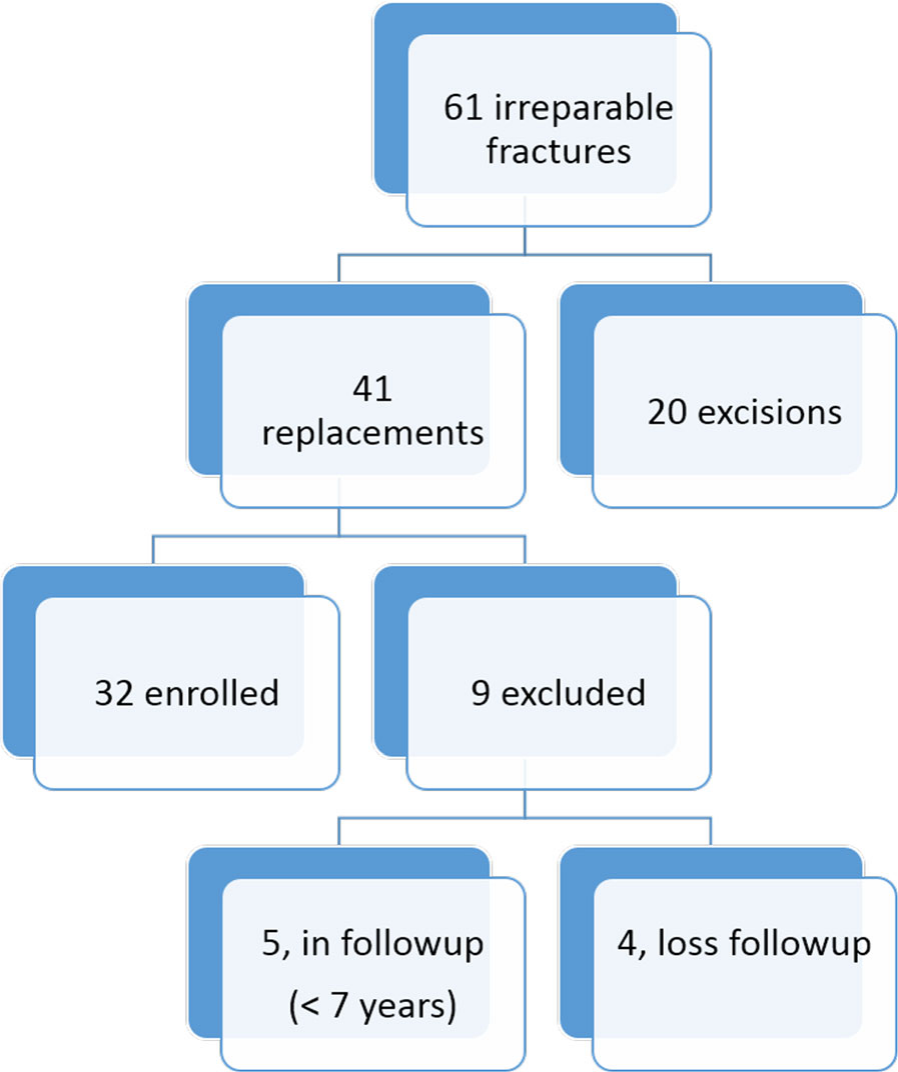
Inclusion and exclusion patients

Between 2004 and 2011, we identified 61 patients from our surgical database who underwent surgery for irreparable radial head fractures. Radial head excision was performed in 20 elbows of 20 patients who failed to respond to nonsurgical treatment for at least 1 month. RHR was performed in the other 41 elbows of 41 patients. All replacement surgeries were preoperatively approved by at least two orthopedic surgeons in our department, and the surgical indication was well documented in the medical records. Because one single type of radial prosthesis was used for all replacement surgery in our institute from 2004 through 2011, this study only enrolled the surgeries performed during this period to avoid implant-selecting bias. Of 41 patients, 32 with more than 7-year follow-up were enrolled in this study, whereas the remaining 9 patients either had short-term follow-up or were lost to follow-up (Fig. 1). All patient and injury characteristics summarized in Table 1. There were 18 men and 14 women with an average of 43.91 ± 13.70 years (range, 14 to 75 years). The right and the left elbows were involved in 17 and 15 patients, respectively. The mean time from trauma to surgery was 10.13 ± 29.21 months (range, 0 to 120 months); 17 patients had acute injuries within 1 month, whereas 15 patients that were referred from other clinics had subacute or chronic injuries. Those referred patients were treated either non-operatively or failed to previous open reduction surgery. The diagnoses were valgus-type injuries (14 patients: 4, acute injury; 10, chronic injury), terrible triad injury (14 patients), Monteggia fracture (3 patients), and supraintercondylar fracture of the distal humerus (1 patient). Previous surgery (1 to 3 times) before replacement surgery was noted in 8 patients. All 32 patients had regular follow-up of more than 2 years. The latest survey was performed at 7 to 15 years postoperatively.

**Table 1:** Patient characteristics

Characteristics	No. (%) of patients
Total	32 (100)
Gender	
Men	18 (56)
Women	14 (44)
Dominant side injured	17 (53)
Associated fractures	
Elbow dislocations	14 (44)
Coronoid fractures	14 (44)
Distal humerus fractures	1 (3)
Proximal ulnar fractures	3 (9)
Ligament repair	
Lateral collateral ligament	14 (44)
Medial collateral ligament	1 (3)
Time from injury to replacement	
< 1 month	17 (53)
≥ 1 month	15 (47)
Radial head replacement	
As a primary surgery	24 (75)
As a revision surgery	8 (25)

### Surgical procedure

All replacement surgeries were performed by a single surgeon. In 28 patients, the radial head was exposed using the posterolateral Kocher approach. The Boyd approach was used in three cases in which a Monteggia fracture was involved. A global approach along the previous surgical scar was used in a patient with concomitant distal humerus nonunion. The Kocher interval between the anconeus and the extensor carpi ulnaris was identified by a thin strip of fat and bluntly dissected into the deep fascia. The deep fascia at this interval was incised, and the muscles were retracted to expose the lateral ligament complex, which was identified by meticulous dissection along the muscular–capsular plane. Complete tear of the lateral ligamentous–capsular structure was found in all patients with terrible triad injury, which was reattached using transosseous and anchor sutures after completion of prosthesis implantation. Medial collateral ligament was explored and repaired in the cases with grade III or more instability on valgus stress test intraoperatively after replacement.

RHR was performed in all patients using a modular uncemented smooth-stem prosthesis (EVOLVE radial head system, Wright Medical Group, Arlington, TN), which consisted of a head segment and a smooth stem with options for neck length. The size of the head segment was intraoperatively determined by reassembling the major fragments of the radial head on a sizing tray by selecting a similar diameter or 1 mm downsized. The stem size was determined after sequential canal reaming and calcar trimming. The total height of implanted prosthesis was adjusted using trial prosthesis and finally determined by a combination of head thickness and neck length with the proximal margin of the head segment reaching or 1 mm distal to the horizontal level of the coronoid tip, which was confirmed by C-arm fluoroscopy during surgery.

### Clinical review

All patients were located through the National Health Insurance Service Register, which contained registration files and original claim data for reimbursement. Patient demographic and surgical data were well documented in the medical records. Chang Gung Institutional review board approval (IRB 201800206B0) was obtained for the review of the medical records and invitation to patients to return for evaluation. Clinical and radiographic evaluations were performed at the latest follow-up by one of the co-authors who had not been involved in the treatment of the patients. One musculoskeletal fellowship-trained radiologist of more than 5 years seniority was also invited to perform radiographic evaluation.

### Functional survey

Pain or soreness around the involved elbow was evaluated using the visual analog scale (VAS), with scores ranging from 0 to 10 points. The Mayo Elbow Performance Score (MEPS) and shortened Disabilities of the Arm, Shoulder, and Hand (quickDASH) score were used for functional evaluation. The MEPS is a widely used performance index for evaluating clinical outcomes of a variety of elbow disorders and shows validated reliability and accuracy for evaluating the treatment results of elbow fractures and dislocation [17]. Being a shortened version of the DASH outcome measure, the quickDASH consists of 11 items (scored 1–5) instead of 30 items as in the DASH questionnaire to evaluate perceived physical function and symptoms in individuals with upper limb musculoskeletal disorders [18].

### Radiographic survey

**Fig. 2:**
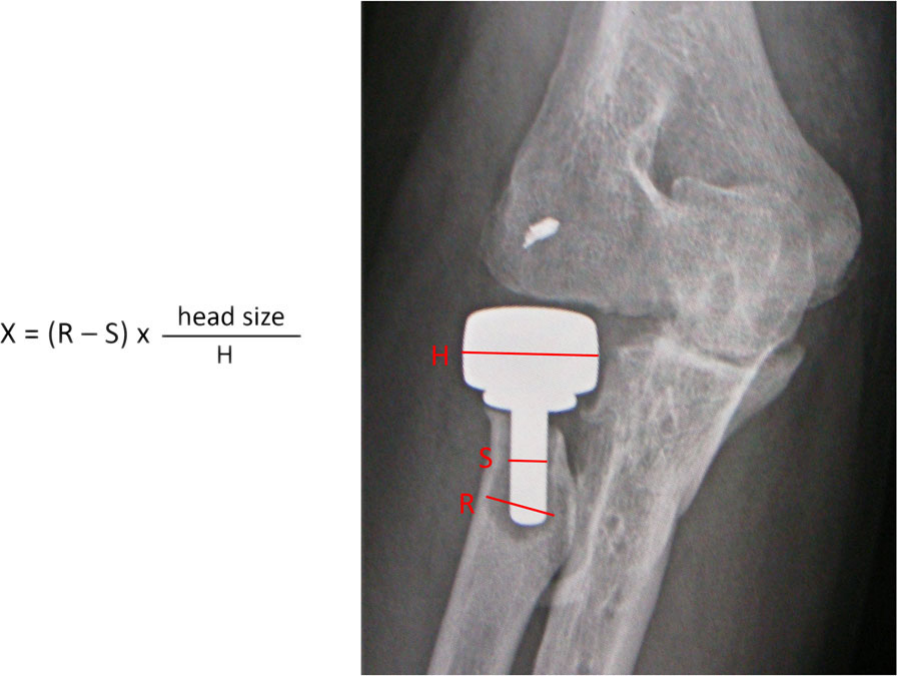
Thickness of radiolucency (*X*) was calculated using the elbow radiograph. R, maximal width of decreased density around the stem. S, width of the stem. H, width of the head. Head size was the diameter of radial head prosthesis according to the surgical record (unit, mm)

Radiographic analysis included the radiolucent area around the prosthesis, presence of osteoarthrosis, and heterotopic ossification. There were two radiographs for each elbow. Anteroposterior view was taken with the elbow in maximal extension and the forearm in maximal supination; lateral view was taken with the elbow in 90° flexion and the forearm in neutral rotation. There were two evaluators who were blind to the patients’ demographics. The sum of maximal width of decreased density around the stem (Fig. 2) was measured from the anteroposterior and lateral views. Radiolucency of one elbow was defined as the average of the measured thickness from the two evaluators.

### Statistical analysis

Descriptive statistics were calculated for key variables. Independent *t* test was used for intergroup comparison. A *p* value < 0.05 was considered significant. The association between mean radiolucency and VAS scores was assessed using Spearman correlation coefficient.

## Results

All 32 patients returned for follow-up at an average of 8.94 ± 1.81 years (range, 7 to 15 years) after RHR. None underwent subsequent revision surgery or removal of the radial head prosthesis.

### Functional outcomes

All patients regained a mean motion arc of 119.4° ± 16.8° (range, 65° to 140°). Extension deficiency, supination, and pronation averaged 4.5° ± 6.5° (range, 0° to 30°), 82.3° ± 7.6° (range, 60° to 90°), and 87° ± 5.8° (range, 60° to 90°), respectively. There were 24 patients (75%) showing residual soreness or pain, around the lateral elbow or proximal forearm. The pain was mild in most patients; only two patients present moderate pain. VAS scores averaged 1.25 ± 1.16 (range, 0 to 5). Eight patients were pain-free. With respect to functional evaluation, MEPS averaged 83.4 ± 13.9 (range, 45 to 100). Excellent, good, and fair results were achieved in 10, 16, and 5 patients, respectively, with 1 patient having poor result. Disability scores based on quickDASH averaged 11.7 ± 13.5 (range, 0 to 50). Intergroup analysis of MEPS and quickDASH scores based on age, gender, injury chronicity, injury complexity, and number of surgeries is summarized in Table 2. Significantly better MEPS was noted in women than in men (*p* = 0.025); however, the difference in quickDASH scores was not significant. With respect to disability, significantly higher quickDASH scores were noted for complex injuries than for simple valgus-type injuries (*p* = 0.020), whereas the difference in MEPS was not significant. Better functional outcomes were found in patients who underwent primary replacement surgery than in those who underwent repeat surgery; the difference was significant for both MEPS (*p* = 0.023) and quickDASH scores (*p* = 0.026). Otherwise, there was no significant difference in intergroup comparison of functional outcomes with respect to age and injury chronicity.

**Table 2:** Intergroup comparison of functional outcomes

Subgroup comparison	No.	MEPS	*p* value	QuickDASH	*p* value
Age (years)					
< 45	18	80 ± 14.35	0.058	14.53 ± 14.34	0.088
≥ 45	14	87.86 ± 12.51		7.95 ± 11.79	
Gender					
Men	18	79.17 ± 13.53	0.025*	14.65 ± 14.06	0.078
Women	14	88.93 ± 12.89		7.79 ± 12.12	
Chronicity					
< 1 month	17	83.53 ± 10.72	0.485	11.10 ± 9.84	0.405
> 1 month	15	83.33 ± 17.29		12.27 ± 17.07	
Injury complexity					
Valgus impact^†^	14	87.5 ± 10.52	0.074	6.17 ± 7.33	0.02*
Rotational injury^‡^	18	80.28 ± 15.67		15.91 ± 15.71	
Numbers of surgery before replacement					
None	24	86.25 ± 10.96	0.023*	9 ± 9.59	0.026*
≥ 1 surgery	8	75 ± 18.9		19.6 ± 20.19	

^†^Valgus impact was valgus impact injury of radial head with or without medial collateral ligament insufficiency

^‡^Rotational injury included terrible triad injury, Monteggia injury, and concomitant distal humerus fracture

*A *p* value of < 0.05 indicated significant difference

### Radiographic analysis

**Fig. 3:**
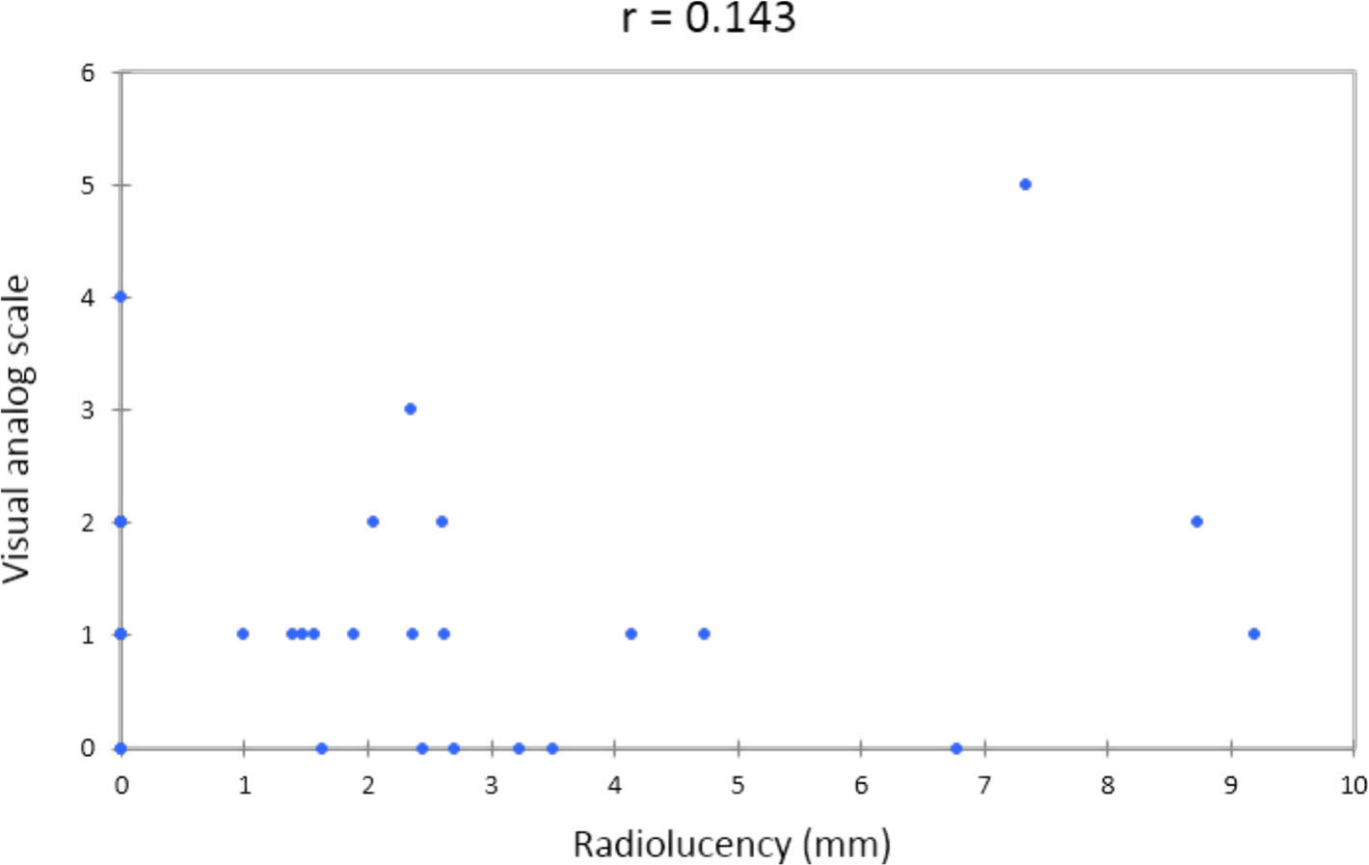
Correlation between measured width of radiolucency (mm). Visual analog scale scores were assessed using Spearman correlation coefficient (*r*)

Elbow radiographs at the latest follow-up were blindly reviewed by both orthopedic and radiologic specialists. Periprosthetic radiolucency was identified and measured in 21 patients (66%) with a mean thickness of 3.53 ± 2.43 mm (range, 1.35 to 8.74 mm). No remarkable radiolucency was noted on the radiographs of the other 11 patients. With respect to the comparison of functional outcomes between patients with and without radiolucency, the *p* values were 0.127 for MEPS and 0.135 for quickDASH scores (Table 3). No significant difference was noted. The Spearman correlation coefficient between the measured width of radiolucency and VAS scores was 0.143 (Fig. 3). The correlation between residual pain and periprosthetic radiolucency was low.

**Table 3:** Functional comparison according to the presentation of radiolucency

	Radiolucency + (*n* = 21)	Radiolucency − (*n* = 11)	*p* value
MEPS^†^	85.48 ± 14.13	79.55 ± 13.31	0.127
quickDASH^‡^	9.53 ± 12.11	15.7 ± 15.6	0.135

^†^*MEPS* Mayo Elbow Performance Score

^‡^quickDASH = shortened Disabilities of the Arm, Shoulder, and Hand

Other radiographic findings included medial heterotopic ossification in 3 patients (9%) and ulnohumeral arthrosis in 2 patients (6%). All of these patients showed a motion range greater than 120°. Pain-free motion was regained in all except 1 patient, who was 1 of 2 patients with ulnohumeral arthrosis and presented with mild pain during vigorous activity. No capitellar erosion was noted.

## Discussion

As the radial head is an important secondary stabilizer of the elbow [19], replacement surgery is advised for patients with unreconstructible radial head fractures and concomitant ligamentous injuries, which call for its secondary stabilizing function. Through the use of a metal spacer to restore elbow articulation, RHR provides immediate stability with encouraging outcomes. Given the prevalence of injuries and age at surgery in the young and active population, long-term results and longevity of prosthesis become a critical issue after implantation. Current series reported different revision rates ranging from 0 to 29%, with no evidence supporting one type of radial head prosthesis over another [20].

The strengths of this study are the long-term follow-up in a single-unit study and the validated clinical outcome measurements. Furthermore, we proposed a reliable and straightforward method for radiolucency measurement based on the real diameter of radial head prosthesis. This measurement not only is useful in case series studies but also can be applied in longitudinal cohort surveys regardless of the image ratio on different radiographs.

In the present study, modular monopolar prosthesis with loose-fit stem was used in 32 patients with a mean follow-up of 8.94 years. Functional and radiographic survey was performed in all patients at 7 to 15 years after replacement surgery. Based on MEPS, 26 achieved good to excellent results. There were 6 patients with fair or poor results including one case of periarticular HO and five cases with delayed replacement (2 primary and 3 revision surgeries). Both MEPS and quickDASH scores were comparable with the midterm results in our previous report and other series. All prostheses survived; none of the 32 patients underwent prosthesis revision or removal. Only few patients had late sequelae such as arthrosis and heterotopic ossification; the presentation was mild. We believe that our study has the longest follow-up to date that demonstrated the surgical outcomes of RHR. Moreover, we analyzed the difference according to patient characteristics. Significantly better MEPS and quickDASH scores were noted in patients who underwent direct RHR than in those who underwent surgical fixation prior to RHR. As recent studies reported superior outcomes of RHR over fracture fixation for complex radial head fractures [14, 15], our findings further supported the strategy of direct RHR not only to facilitate early motion but also to minimize late sequelae.

Periprosthetic radiolucency was commonly observed in radial head prosthesis with loose-fit stem. Several studies reported the relationship between radiographic analysis and clinical survey while being limited to short-term to midterm survey [21, 22]. With radiographic analysis at 7 to 15 years after replacement surgery, two third of our patients showed periprosthetic radiolucency. The incidence in our study was no greater than that in previous short-term to midterm studies, but the mean thickness of radiolucency (mean, 3.53 mm; range, 1.35 to 8.74 mm) in our study was much greater than that (median, 1.67 mm; range, 0.08 to 1.95 mm) in a previous report [21]. With respect to the comparison of functional outcomes between patients with and without radiolucency, no significant difference was found. Although as many as three fourths of patients had residual pain, the correlation between radiolucent size and pain scores was low. Radial head prostheses with loose-fit stem were basically designed to restore elbow kinematics while providing enough mobility to adapt to the complex anatomy by increasing modularity and offering greater range of size options [23]. Periprosthetic radiolucency was thus commonly observed with progression. Based on our investigation, it seemed that increased radiolucency did not compromise long-term outcomes; however, high percentage of residual pain and potential late progression should caution the surgeon to ensure more accurate performance regarding prosthesis sizing and application.

The weaknesses of our study were that it retrospectively reviewed a consecutive series with heterogeneous cohort and lacked a control group. In addition, the exclusion of 9 patients because of follow-up loss and insufficient follow-up may have substantial effect on outcome analysis. Although we aimed to perform a long-term study, the overriding benefits of longevity survey may be offset by the huge discrepancy in follow-up duration from 7 to 15 years among the 32 patients. Finally, some patients were not regularly followed up, and the final survey was based only on the latest visit.

## Conclusion

RHR using a metallic modular smooth-stem prosthesis is a feasible treatment option for patients with unreconstructible radial head fractures with or without associated osseous and soft tissue injuries; sustained clinical outcomes were reported at 7 to 15 years of follow-up. No patients underwent prosthesis revision or removal. Significantly better functional outcomes were found when replacement surgery served as the primary surgery instead of late salvage after repeat surgery. Periprosthetic radiolucency was not correlated with functional or pain scores, whereas residual pain and potential late progression in long-term follow-up may caution the surgeons to perform surgical procedures elaborately and warrant meticulous survey.

## Abbreviations

MEPS: Mayo Elbow Performance Score
quickDASH: Shortened Disabilities of the Arm, Shoulder, and Hand
RHR: Radial head replacement
VAS: Visual analog scale
